# Reduction in deep sternal wound infection with use of a peristernal cable-tie closure system

**DOI:** 10.1186/1749-8090-10-S1-A213

**Published:** 2015-12-16

**Authors:** Meghan M Stelly, Charles B Rodning, Terry C Stelly

**Affiliations:** 1 Cardiothoracic and Vascular Surgical Associates, Mobile, AL, USA; 2 Department of Surgery, College of Medicine, Medical Center, University of South Alabama, Mobile, AL, USA

## Background/Introduction

Deep sternal wound infections (DSWI) are a rare but serious complication after median sternotomy.

## Aims/Objectives

We evaluated postoperative outcomes associated with two sternal-closure techniques.

## Method

Methods and outcomes of sternal closure were reviewed in consecutive patients undergoing a variety of cardiothoracic surgical procedures. Sternal closure in the historical control group was performed using trans-sternal, stainless-steel wire sutures; subsequent patients were closed using wire sutures together with a novel, peristernal cable-tie closure system to reinforce the corpus sterni. Perioperative care was standardized between groups.

## Results

Between July 2010 and July 2014, 609 consecutive adult patients underwent sternal closure following open median sternotomy at a single hospital in Mobile, Alabama. Sternal closure was accomplished with wire sutures in the first 309 patients and with cable-tie reinforcement in the subsequent 300 patients. One author performed 71.8% (222/309) of the wire suture procedures and 92.7% (278/300) of the cable-tie procedures; the others were performed by a single other partner in his practice. Baseline characteristics were comparable between groups, except that the cable-tie group exhibited greater preoperative comorbidity. Mean body mass index was comparable between groups (30.2 ± 6.6 kg/m2 wire suture versus 30.5 ± 7.7 cable-tie, p = 0.568). DSWI occurred in 2.6% (8/309) patients in the wire-suture group, whereas no DSWI were observed in the cable tie group (p = 0.008). Analysis of STS data shows the authors' rate of DSWI to be 0.0% in each of the years from 2012 - 2014 (corresponding with adoption of the cable-tie system), compared to an overall institutional rate of 0.2% and an overall STS database rate of 0.3% for 2014.

## Discussion/Conclusion

The peristernal cable-tie system was a simple and reliable method for sternal closure after open median sternotomy, and was associated with a reduced risk of deep sternal wound infection, even in an obese and comorbid population.

